# Images: Malignant right coronary artery - 64-slice CTA

**DOI:** 10.4103/0971-3026.40293

**Published:** 2008-05

**Authors:** RS Rama Krishnan, Vandana V Marwah, Tanuj Gupta, Arjun Kalyanpur

**Affiliations:** Department of Radiology, Narayana Hrudayalaya, Bommasandra Industrial Area, Bangalore, India; 1 Teleradiology Solutions, Plot No. 7G, Opp. Graphite and Department of Radiology, Narayana Hrudayalaya, Bangalore, India

## Case History

An asymptomatic 48-year-old man came to our institute for routine cardiac evaluation. Electrocardiogram, echocardiogram, and treadmill exercise testing were within normal limits. The patient opted for a CT angiogram (CTA) of the coronary arteries and was subsequently referred to our department. The angiogram was performed on a Light speed VCT (Wipro GE, Bangalore) using a standard protocol.

**Figure 1:**
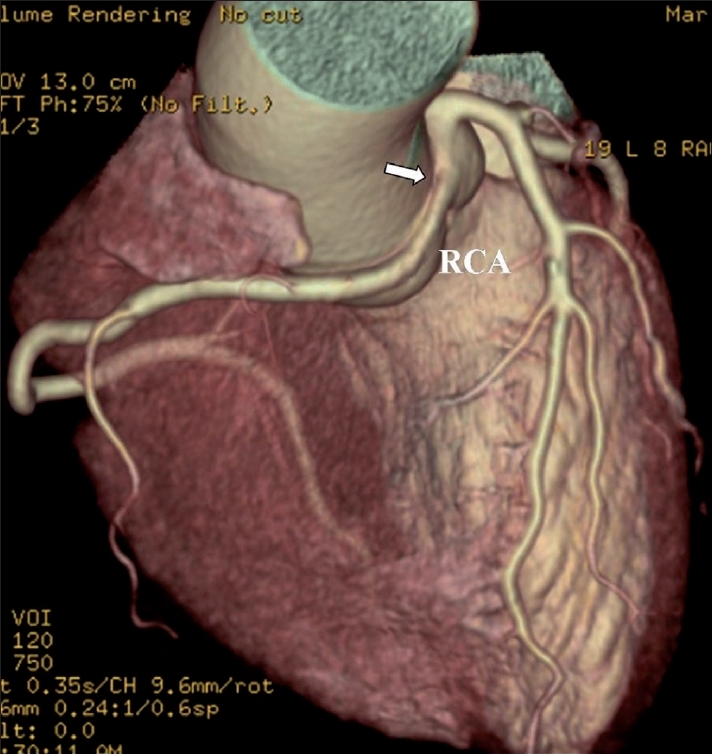
Volume-rendered image showing an anomalous RCA (arrow) originating from the left coronary sinus and coursing between the aortic root and main pulmonary artery

**Figure 2:**
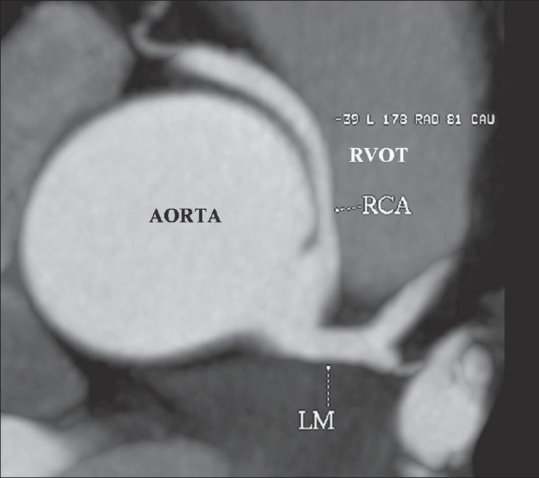
Axial maximum-intensity projection image showing the origin of the RCA from the left aortic sinus and its course between the RVOT and aorta. The compression of the RCA during its interarterial course is well appreciated. The normal origin of the left main coronary artery is also seen

**Figure 3:**
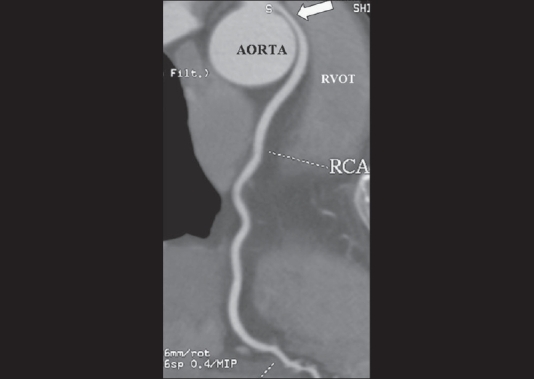
Coronal MPR image showing the compression of the proximal RCA (arrow) as it courses between the RVOT and aorta

The study revealed an anomalous right coronary artery (RCA) originating from the left coronary sinus and coursing between the aortic root and main pulmonary artery [Figure 1]. Narrowing of the proximal RCA was seen [Figures 2, 3] as it passed between the aorta and the right ventricular outflow tract.

## Discussion

The findings described above are characteristic of a malignant RCA. It is called “malignant” because the RCA can undergo compression between the aorta and the pulmonary trunk, especially during exercise, and this carries the risk of sudden cardiac death.[1]

Coronary artery anomalies occur in about 1% of patients undergoing cardiac catheterization. Approximately 20% of coronary artery anomalies produce life-threatening symptoms, such as arrhythmias and syncope, or may cause myocardial infarction or sudden death.[2] In particular, an anomalous vessel that crosses between the aorta and the main pulmonary artery, either a left coronary artery originating from the right sinus or a RCA emanating from left sinus, may be associated with a poorer outcome.[3] The causes of ischemia in patients with anomalous origins of the RCA may include: 1) narrowing of a slit-like, small ostium; 2) intramural course of the proximal portion of the RCA, within the aortic wall; in addition to 3) compression of the RCA as it courses between the pulmonary artery and the ascending aorta; 4) kinking and bending of the anomalous RCA; and 5) spasm of the proximal portion of the RCA.[3]

At catheter angiography, the precise course of the anomalous vessel may be difficult to delineate, because its complex three-dimensional geometry is displayed in two dimensions fluoroscopically.[2] Difficulties with catheter engagement of the anomalous vessel can lead to the erroneous assumption that the vessel is occluded.[3]

Multi-detector CT (MDCT) with its cross-sectional imaging capability is a viable noninvasive modality for delineating coronary artery anomalies, particularly if findings at coronary angiography are equivocal.[3] MDCT is rapidly emerging as a credible alternative for the visualization of coronary anomalies.[4] The drawbacks of this technique include the need for intravenous infusion of contrast agent and exposure to ionizing radiation.[5]
